# Show what you know and deal with stress yourself: a qualitative interview study of medical interns’ perceptions of stress and gender

**DOI:** 10.1186/1472-6920-14-96

**Published:** 2014-05-17

**Authors:** Petra Verdonk, Viktoria Räntzsch, Remko de Vries, Inge Houkes

**Affiliations:** 1 Department of Medical Humanities, VU University Medical Centre, EMGO Institute for Health and Care Research, School of Medical Sciences, Amsterdam, The Netherlands; 2 Clinipace AG, Volketswil, Switzerland; 3 Cormel IT Services, Sittard, The Netherlands; 4 Department of Social Medicine, Maastricht University, School Caphri, Maastricht, The Netherlands

**Keywords:** Gender, Stress, Interns, Rotations, Medical education, Coping

## Abstract

**Background:**

Medical students report high stress levels and in particular, the clinical phase is a demanding one. The field of medicine is still described as having a patriarchal culture which favors aspects like a physicians’ perceived certainty and rationalism. Also, the Effort-Recovery Model explains stress as coming from a discrepancy between job demands, job control, and perceived work potential. Gendered differences in stress are reported, but not much is known about medical interns’ perceptions of how gender plays in relation to stress. The aim of this study is to explore how medical interns experience and cope with stress, as well as how they reflect on the gendered aspects of stress.

**Methods:**

In order to do this, we have performed a qualitative study. In 2010–2011, semi-structured qualitative interviews were conducted with seventeen medical interns across all three years of the Masters programme (6 male, 11 female) at a Dutch medical school. The interview guide is based on gender theory, the Effort-Recovery Model, and empirical literature. Transcribed interviews have been analyzed thematically.

**Results:**

First, stress mainly evolves from having to prove one’s self and show off competencies and motivation (“Show What You Know…”). Second, interns seek own solutions for handling stress because it is not open for discussion (… “And Deal With Stress Yourself”). Patient encounters are a source of pride and satisfaction rather than a source of stress. But interns report having to present themselves as ‘professional and self-confident’, remaining silent about experiencing stress. Female students are perceived to have more stress and to study harder in order to live up to expectations.

**Conclusions:**

The implicit message interns hear is to remain silent about insecurities and stress, and, in particular, female students might face disadvantages. Students who feel less able to manifest the ‘masculine protest’ may benefit from a culture that embraces more collaborative styles, such as having open conversation about stress.

## Background

Mental health problems such as high stress levels and substance abuse are highly prevalent among physicians [1-4]. Medical students show high stress levels early on in their studies [5-8], and the transition from the preclinical to the clinical phase is particularly demanding [6,9,10]. However, the medical profession seems uninterested in its members’ mental health [7]. In addition, professional norms in medicine are said to be more in line with masculinist gender norms, largely influenced by competition, than with feminine norms, which focus on open conversation and empathy [11-14]. Stress-related disorders such as anxiety and depression are more prevalent among women e.g. [15], and a higher prevalence of work-related stress is found among highly educated women than their male counterparts. This is found already between those of 15–25 years of age [16]. We have conducted a qualitative interview study to explore how medical interns in the Netherlands experience and cope with stress as well as how they reflect upon gender in this process.

### Medical education in the Netherlands

As a consequence of the Bologna Declaration of 1999, which aims towards, amongst other things, recognition of degrees [17], medical education in Europe is moving toward more harmonization. Therefore, a unified structure with separate undergraduate and graduate programmes has been set up in the Netherlands. Generally, curricula are vertically integrated. Medical schools teach basic medical sciences, basic communication and clinical skills, and professionalism as applied to medicine in the bachelor programme, whereas in the master programme clinical and communication skills are developed together with an in-depth understanding of the basic sciences [17]. In the Netherlands, local differences between the eight medical schools exist in, for instance, educational approaches (e.g., problem-based learning versus traditional ways of teaching), and in the student population (as schools in the western part of the country attract more cultural minority students with migrant backgrounds). Despite these differences, all eight schools fulfill the same final objectives for medical education as defined in the Dutch *Framework 2009*[18], and the female:male ratio of the Dutch student population is on average 2:1. This ‘feminization’ of medical education is also reported elsewhere, such as in the Nordic countries, the United Kingdom, the United States, Canada and Australia [19]. This gender ratio in medical education is related to girls’ higher interest in medicine and to their higher academic achievements in secondary education, a selection criteria for medical education in the Netherlands, being higher grades associated with a relatively lower chance of dropping out [20].

### Stress among medical students

Stress can be defined as the experience of a discrepancy between perceived demands and perceived resources. ‘Fairly strong’ stress and a high prevalence of mental health problems (17-33%) are found among medical students [8,21-23]. Stress levels increase during medical education and rise further throughout residency [7,21,23-25]. Post-mortem human dissection is highly reported as a stressor among first year students. Other sources of high and more chronic stress are the increasing workload [25], and concerns about academic performance in conjunction with lacking leisure time, particularly during the clinical phase, when professional knowledge and skills are put to the test [6]. Communicating with sick and dying patients and their families can also be a source of stress [9].

In the clinical phase, students move through the specialties by learning-on-the-job. Each new rotation requires unique medical knowledge and a set of skills, which often highlights students’ deficiencies more than their progress and competencies [8]. Students in their clinical years often feel inadequately prepared [10], and transition courses are suggested [26]. Furthermore, many students feel insecure about the expectations of the faculty and clinical staff regarding their performance. The majority of students wish for more clear descriptions of the requirements regarding professional behavior and task performance, and for more sufficient supervision [26,27]. Another major issue in medical education is the abuse faced by students and specialists in academic hospitals, such as verbal abuse or excessive workloads [8,28-30]. Female students in particular are prone to sexual harassment by fellow students, supervisors and patients [11,31-33]. Abuse affects students’ self-confidence in regards to clinical skills and mental health problems [34]. Although stressful internships are considered a ‘rite de passage’ strengthening young doctors, high stress levels among physicians show that stress is not a transient phenomenon [3,7]. Besides, gender differences occur.

### Theoretical and empirical underpinnings

#### Gender theory

People make sense of the world by dividing it into categories, including those of gender [35]. The concept of ‘gender’ is commonly used to refer to the social aspects of being a man or a woman, differentiated from their biological aspects, for which the concept of ‘sex’ is used [36]. Gender is used to organize societies and is incorporated within institutions and individuals in many ways. Gender socialization refers to the developing of a male or female identity by internalizing certain roles, tasks and characteristics assigned to these labels. The categories of men and women are not only assigned different characteristics, as they are considered to be mutually exclusive, but these categories are also ordered hierarchically in power relations [35]. Despite gender ideology that prescribes that women are feminine and not masculine and that men are masculine and not feminine, men and women do assign both aspects of femininity and of masculinity to themselves [37]. Femininity refers to what are often considered desirable traits for women, such as being shy and caring, and masculinity to what are desirable traits for men, such as being athletic and rational [38]. Higher status is assigned to men and to masculinity, and those of higher status are assumed to be competent. Those of lower status are considered to carry the ‘burden of proof’, needing to show that they are competent [14,39]. But gender is not only a characteristic or trait. Gender is also a lived experience to which processes of gender or ‘doing gender’ are relevant [35,40]. Gender is both a material and a cultural phenomenon; men and women are socialized so that gender becomes an individual’s identity, which is maintained in daily interactions and through performative actions [35]. As gender is embedded in individual identities, masculinity and femininity apply to both men and women; gender is *relational* because it is actively developed and maintained through interaction; and last but not least, gender is integrated into arenas at the structural level, such as in the healthcare system [36].

A patriarchal culture is often said to exist by a pattern of ‘male dominance’, and, as an institution, medicine has long been dominated by men. For instance, despite the feminization of the medical profession in many countries, there is still an unequal distribution of men and women over specialties and in medical leadership [19]. However, calling it a patriarchal culture does not necessarily refer only to the dominance of male doctors but, rather it refers to a culture that favors heroism, rationalism, certainty, distance, and objectification at the expense of more collaborative approaches [14]. Bleakley calls this culture of distancing oneself ‘the masculine protest’. Since physicians see so much suffering, this ‘masculine protest’ helps physicians to protect themselves against admitting to uncertainty, vulnerability and feelings of powerlessness. And although distancing oneself is a widely adopted coping style for doctors, it may come at a psychological price, such as experiencing stress or a decline in empathy. Furthermore, the ‘masculine protest’ does not work for all physicians, and the consequences of failed protesting can be psychological health complaints such as higher burnout rates, substance abuse and depression [14]. Hidden rules for appropriate behaviors of male and female physicians are also embedded within medical culture [19]. For instance, distancing oneself may for instance be a more desirable trait for men than for women, whereas admitting to vulnerability may be more desirable for women than for men.

Evidence suggests that depersonalization or depressive symptoms are not necessarily related to direct contact with patients and patient suffering, but rather that other aspects of the medical working environment are risk factors. In particular, working conditions such as excessive workload, lack of support from supervisors and harassment are determinants of physicians’ ill mental health e.g. [41,42]. These factors are also found to play a role in medical students’ reports of stress [8]. Furthermore, they resemble the profile of a patriarchal culture, which favors, for instance, competitiveness over collaboration.

#### Effort-recovery model

In the Effort-Recovery Model, the development of stress is explained by a discrepancy between: (a) experienced job demands; (b) experienced job control; and (c) experienced work potential [43,44]. Job demands are conditions such as time pressure, emotional or physical demands at work. Job control refers to decision latitude, such as the ability to decide when and how a particular task is executed. Work potential, also incorporating individual factors, entails aspects such as knowledge and skills, willingness or motivation to meet demands, and coping style. When, for instance, job demands are perceived as high and individual skills are estimated to be insufficient, then these three dimensions are perceived as being out of balance. Generally, recovery occurs by temporarily ceasing to put energy into a task, e.g. during a break or after work by restoring depleted resources (by means of sleeping, spending time with friends or doing sports), or by increasing work potential through learning and developing skills/increasing competencies. But for medical interns, each rotation requires a new set of skills and students may have little time to rest [6,8].

#### Gender and stress among medical students

Medical education is a lived experience that constructs professional identities with a strong defense against admitting vulnerabilities and uncertainties [14]. Thus, students have to learn to deal with an organizational culture during their internships that may well be related to the feelings of stress that this type of culture originally had aimed to prevent. Internships in particular are meant to support the transition from student identities to professional identities. Not only does gender play a role in medical students’ own experiences, but students may also perceive how other students, male and female, deal with stressors during their internships, or, how gender and stress are ‘done’.

Some studies found no or small gender differences in stress symptoms among medical students and physicians [1,3,21,22,45,46]. In other studies, female physicians and female students report burnout, stress, anxiety and depression more often than men [12,13,41,47]. Female medical students worry more often about their academic performance and career decisions than men do [12]. In an earlier study, female medical students rated stressful life events more negatively and reported less personal achievements than men [48]. An Israeli longitudinal study found a higher vulnerability to anxiety caused by the pressure of academic and medical competencies among female medical students [49]. Even when objectively, female medical students’ academic performances are higher [50], gender bias is exposed in performance evaluations [51]. Women’s intellect (more often defined as ‘sensitive’) was given less credit than comparable males’ intellect (more often ‘quick learners’) [51]. Hence, women’s abilities may be questioned more than men’s by their assessors, peers and themselves. In general, women tend to underestimate their abilities whereas men are more likely to overestimate their abilities and performances [52]. Observers perceive female medical students as significantly less confident than male students [53]. Female students may be more sensitive to other people’s emotions and reflect more about their own reactions and performances [12,54]. Generally, women are more likely to engage in rumination, or passively focusing on one’s symptoms of distress and on their possible causes and consequences [55], amplifying chronic strain [56].

Physicians and medical students report high stress levels and gendered experiences and responses to stress seem to occur. In particular, stress is related to factors in the work environment and people’s perceptions of these factors. Distancing oneself and, thus, trying to fit into a ‘patriarchal culture’ (for instance by not admitting to vulnerability), seems an important characteristic of physicians’ professional roles, and thus students must be taught and learn to internalize this professional standard. And although medical education is associated with high stress levels already early on, ‘doing doctor’ is in particular learned from ‘lived experience’ during rotations. From the foregone, reported differences between male and female physicians suggest that gendered aspects play roles in how medical students perceive and learn to deal with work stressors and the work environment. So far, medical students’ reflections on gendered aspects in stress have not been studied. Hence, our research question is: “How do medical interns experience and cope with stress and how do they reflect on gendered aspects in stress?”

## Methods

### Participants

Since 2006, Maastricht University has offered separate Bachelors’ and Masters’ programmes with a problem-based learning approach [57]. During the three clinical years, students rotate through internships in different wards, from the academic hospital to peripheral hospitals in the region (involved in the education of interns during the clinical phase as well).

In 2010–2011, seventeen interviews were conducted to investigate Dutch medical interns’ experiences of stress. We have aimed for purposive sampling to maximize the richness, depth and variability of the data. We have also aimed to select participants that could provide the most informative data while providing different perspectives on the topic, based on age, gender, master year and relationship status [58].

In order to recruit interviewees, originally announcements were distributed. But this yielded no response. Therefore, students were directly approached through personal contacts, after education meetings and through snowballing. This turned our sampling strategy more into one of convenience, but with the required diversity among our interviewees, in for instance gender and master year. Students were invited to participate in a study of stress among medical interns. The aim of this study was explained to the students as to gain insight into how they experience stress, factors leading to stress and its consequences, with the ultimate goal of providing recommendations for improving the medical curriculum in regard to stress prevention. Furthermore, we explained our interest in gendered aspects to these students. We emphasized that the students were selected as possible participants in this study because they fit the characteristics of the proposed research population. Interviewees were not known to the interviewers.

Table 1 provides an overview of the participants’ characteristics. None of the participants reported to have children. All participants had Dutch nationality. Six interns were male, and 11 were female. This reflects the gender ratio in Dutch medical education, where more than two thirds of the medical student population is female.

**Table 1:** Participant information

***Participant***	***Gender***	***Age***	***Year of Education***	***Marital status***
*1*	Female	24	6^th^ year student	Single
*2*	Male	23	5^th^ year student	Relationship
*3*	Male	24	4^th^ year student	Single
*4*	Female	22	4^th^ year student	Single
*5*	Female	23	5^th^ year student	Single
*6*	Female	23	5^th^ year student	Single
*7*	Female	31	5^th^ year student	Single
*8*	Female	24	5^th^ year student	Relationship
*9*	Male	24	6^th^ year student	Relationship
*10*	Female	22	4^th^ year student	Single
*11*	Female	22	5^th^ year student	Single
*12*	Female	23	5^th^ year student	Single
*13*	Male	23	5^th^ year student	Single
*14*	Male	23	4^th^ year student	Relationship
*15*	Female	22	4^th^ year student	Relationship
*16*	Female	22	5^th^ year student	Single
*17*	Male	23	5^th^ year student	Single

### Interview guide

Prior to the subject interviews, we interviewed the Director of Education to gain an understanding of circumstances at Maastricht University with particular regard to medical internships. A topic list (see Table 2) was developed to guide the semi-structured interviews [59].

**Table 2:** Interview topic list

General introduction	Information, informed consent, confidentiality, duration, demographics
Impressions of the internship	Short description, expectations and reality check, choosing medicine
Stress and its determinants	A typical ‘internship’ day, (dis)likes, stressfulness, possible changes, stress as a physician, what are stressful situations and challenges (examples), work load and hours
Consequences of stress	Well-being, changes in (private) life, changes in attitude towards profession, feelings about future
Preclinical education	Preparation for internships
Coping	How do you cope with stress, strategies, how effective, social support, role of supervisors and staff, perceptions of coping with stress among medical staff
Gender aspects	Does being (fe)male play a role, gender differences in patient encounters, differences in treatment by supervisors or patients, social comparison of own performance, what is it like to be (fe)male in the medical profession, how is the fit between own values and hospital/medical culture
Topics for improvement	What would you like to (see) change(d)?
Reflection and additions	How about your choice for medicine

Based on gender theory, the Effort-Recovery Model and empirical literature, in the interviews special attention was paid to stress experiences and coping, how interviewees perceived stress among other interns and supervisors, and whether and how they perceived gender aspects playing a role. For instance, the Effort-Recovery Model was incorporated in questions about work load, hours, stressful situations and challenges (effort), and in how students cope with stress, how they experience support from supervisors (work potential), and what the consequences of stress are in terms of their well-being and changes in their private lives (recovery, or cumulation). In regard to gender theory, questions were asked about how students perceive gender differences in patient encounters and treatment by supervisors, what it is like to be (fe)male in the medical profession and whether they experience a fit between their own values and the hospital/medical culture (see interview guide Table 2).

Interviews were tape-recorded and the interviewers took notes during the interviews. Under Dutch legislation ethical approval is not required, but informed consent forms were signed. The interviewer of the first nine interviews was a female Public Health Masters student. Her interviews were conducted in English because of her German background, and she focused particularly on students’ perceptions of gendered aspects. The second interviewer was a Dutch male Public Health Masters student and he focused more on experiences with supervisors. His eight interviews were held in Dutch.

### Data analysis

Researcher triangulation was applied to base coding and analytical decisions on convergent validation, by discussing coding, clustering, and analyzing among the researchers [58,60]. All interviews were transcribed verbatim. Transcripts were read multiple times in order to get a feeling for the depth of the data and to collect and discuss ideas that came up while reading the data. First, we hand-coded interviews 1–9 (manifest content). We used an interpretative approach by analyzing thematically to identify patterns and categories in the data [59-61]. Data analysis was as inductive as possible, and emergent themes rather than theoretical concepts, lead the analytical process. Analyzing the data in a thematic way was done by the following six constructive steps. First, the first and second author familiarized themselves with the data. This means reading the transcripts over and over again to get a feeling for what the interviewees were actually saying and to get an initial understanding of potentially interesting patterns. The second step was to actually collect the ideas that came up while reading the data. This involved the creation of initial codes out of the raw data by the second author through identifying features of the data and organizing the data into meaningful groups, and discussing the codes with the first author. We negotiated the codes until agreements were reached, but generally there was a high level of agreement. Codes were added to chunks of text or sentences, for instance; personal adjustments and growth, insecurity of not knowing everything, doubting, or ruminating (see an example in Table 3). We coded as many potential patterns as possible to have a rich basis for searching for the actual themes. In the third phase, the different codes were analyzed in order to combine codes in overarching clusters. Thus, categories were constructed by combining codes (axial coding). Initial clusters were for instance working hard, professional skills and knowledge, dealing with expectations, or coping with stress. In the next step, the created themes and sub-themes were reviewed and checked for their validation. For this purpose the data had to be reviewed with the aim of checking whether the themes ‘work’ in relation to the data and if new codes had to be created. In phase five of the thematic analysis, the themes were further refined and themes were analyzed by going back to the data extracts that were put under a cluster of codes and by identifying what was of interest about them and why. Finally, we connected the themes to the data (latent content) [60,61]. The second series of interviews by the third author took place after these initial categories were established. We used interviews 10–17 as a member check to corroborate or falsify our former findings, and compared and adjusted the content accordingly. The second series confirmed and enriched the results of the first series, particularly in regard to students’ perception of supervisor stress, and data saturation, meaning the point at which new information produces little to no change to the codebook, was achieved [62]. Guest et al. conducted an experiment with data saturation and variability. In their study, data saturation occurred for the most part after analyzing 12 interviews in a relatively homogenous population and with similar sets of questions [62]. In our study, the second series hardly added more information although it did provide more nuances. Similarly, our study took place among a homogenous group and a similar interview guide was used. Hence, we conclude that data saturation was achieved. In the results section we present two major themes that reflect the interviewees’ experiences and stories. In Table 3, we provide an example of how we developed codes, categories, clusters and final themes. The study adheres to the RATS guidelines for reporting qualitative studies (http://www.biomedcentral.com/authors/rats, accessed 26 April 2014).

**Table 3:** Data analysis: examples of codes, categories, clusters and themes

**Codes (in bold in overlapping categories)**	**Overlapping categories**	**Combined in cluster**	**Broken down in themes**
**1. Perceptions of internship**	Proving professional skills and knowledge		**Show What You Know…, e.g.**
**2. Stressful thinking**			- Having to prove yourself
3. Perceptions of stress - personality			- Prove your motivation
**4. A typical day**			- Doubting; women perceived to doubt more
**5. Motivation**			- Being noticed
6. Insecurity of not knowing everything/expectations			- Work really hard as implicit message
7. Rumination			
8. Time pressure/workload			
9. Doubting			
**10. Taking work ‘home’/not being able to ‘let go’**			
**11. Planning**			
12. Prove yourself		**Dealing with expectations**	
**1. Perceptions of internship**	Working hard		**… And Deal With Stress Yourself, e.g.**
**2. Stressful thinking**			- Stressful thinking
3. Perceptions of stress - personality			- Individual differences
**4. A typical day**			- Work really hard increases stress
**5. Motivation**			- Taking work home/not being able to ‘let go’
6. Insecurity of not knowing everything/expectations			- Feel and maintain the motivation
7. Rumination			- Planning, goal setting
8. Time pressure/workload			
9. Doubting			
**10. Taking work ‘home’/not being able to ‘let go’**			
**11. Planning**			
12. Prove yourself			

## Results

Two major themes emerged about how students experience and cope with stress. These are elaborated below.

*Show What You Know…*

In order to convince themselves, supervisors and colleagues that they can fulfill the job, students feel they must be prepared to continuously show off their competencies and motivation:

*‘They* [supervisors] *don’t have to make a lot of effort and while you always have to make a lot of effort every time again to show that you’re motivated, to show them that you know stuff and I am motivated, but it’s quite stressful to always show that, it can be quite exhausting.’ (S5, f, 23 yrs, 5*^*th*^*year)*

Male and female medical interns follow the general patterns of gender differences: female interns are perceived to have more stress in relation to having to prove themselves.

*‘I think it’s hard for women to leave things, to just let them go and men can do that better. And I think women want to do things for 100% or 1000% and men just do it, it doesn’t matter, 70% is enough. I think women have higher expectations of themselves.’ (S8, f, 23 yrs, 5*^*th*^*year)*

But female students also report having to work harder in order to come across convincingly. Male interns seem, but definitely are perceived to be, more relaxed and self-confident about their clinical performance and the impressions they make on the wards.

*‘Men are better in organizing things, time management, and women, eh, I do want to do a lot things at once. It’s a bit chaotic for me. And I think they really deal with stress better.* [I: Why?] *Because they can organize better, they also, I don’t know. Because they also don’t show it, to me at least. What I also see is that the girls are more likely to complain than the boys’. (S1, f, 24 yrs, 6*^*th*^*year)*

Generally, students think that the more you know, the better a doctor you are, but possessing knowledge is not enough. For good grades and future careers, students must ensure being noticed by supervisors, and thus, compete:

*‘If you want to have a good grade, then you need to get noticed. So they* [the supervisors] *know ‘Oh, she’s smart’ or ‘Oh, she noticed a lot of things’ or ‘Oh, she knows all about it’. But if you don’t say anything, then they think ‘Oh well, she’s just there or she’s not there, it doesn’t matter!’ That’s a tough world’. (S1, f, 24 yrs, 6*^*th*^*year)*

On the wards, interns often feel like ‘nobodies’ or at most anonymous bodies, for instance because supervisors explain to the patient that ‘the intern’ will take it further. The internships fuel competition between students to prove that they are ‘somebody’. But there are different ways of ‘standing out’. This male student mentions how pretty female students sometimes receive the benefit of the doubt from supervisors:

*‘In general, not many* [gender differences]*, but occasionally with attractive female interns I have a feeling that some doctors maintain different standards. […] More positive feedback for similar work than other people’. (S17, m, 23 yrs, 5*^*th*^*year)*

And although such gender bias benefits these female students’ grades, they are also more often noticed in ways that are less congruent with the physician role. Female students experience that women are granted less credibility, for instance by patients who sometimes think they are nurses or when supervisors ask about boyfriends.

*‘I believe that patients approach women differently. You have to prove yourself more as a woman. They do not trust you as quickly as they trust men and think that you are a nurse’. (S16, f, 22 yrs, 5*^*th*^*year)*

Furthermore, competition seems to cause self-doubt among female students, whereas for male students competition may be more stimulating:

*‘When you start to work in the hospital you are at the bottom of the ladder. You need to climb up and prove yourself, prove that you’re a good doctor, that you know a lot of things and I am not sure if I really want to do that or whether I’m capable’. (S1, f, 24 yrs, 6*^*th*^*year)*

*‘When I am the one who knows less than others, it feels uncomfortable, but it is also a stimulus’. (S17, m, 23 yrs, 5*^*th*^*year)*

Ambiguous demands raise stress. Interns must try to figure out what is expected of them, as sometimes they are given responsibilities above their capabilities and sometimes they hardly get the opportunity to show what they know. The expectations as experienced by the students, incorporate many implicitly expected attitudes. One message is however clear: work hard. Working hard and long hours is perceived as stressful. Yet, they do not find this work ethos problematic, but rather a necessary attitude of physicians. They adapt to what they observe as appropriate behavior because it benefits patients:

*‘There is this culture of working hard, making long days and that this is just normal. But you also have to see that it is difficult to change that. And I don’t know if that would be the best for the patient, I mean doctors who are working long days and always being there for their patients mostly are very good doctors’. (S5, f, 23 yrs, 5*^*th*^*year)*

The interviewees are aware of the high expectations they must live up to during their internships, explicit in knowledge and skills as well as implicit in attitude and motivation.

*… And Deal With Stress Yourself*

Some students try to live up to all the expectations by studying hard to ensure a good academic and clinical performance. For some this works well, but for others, more often female students according to the interviewees, this strategy of working harder increases their stress levels which in turn seems to raise self-doubt. Other students mention that they had lowered their expectations in order to combine study with other life domains and to diminish stress. This interviewee realized that she cannot and will not live up to the high expectations:

*‘I think in the beginning I had a lot more stress, because I thought it would be important to get good grades and prove myself but now I am also satisfied with just passing. When I have the feeling I did enough, this is what I am capable of, I am satisfied. And if they don’t think this is enough, then that’s their opinion’. (S1, f, 24 yrs, 6*^*th*^*year)*

A female student reports how her motivation was lowered when she realized that work in the hospital did not match her expectations of what it was like to be a physician. Nevertheless, most interviewees said they enjoy working with patients; they receive immediate feedback on their performance from the patients, as most are grateful for getting help. As such, patient care is rather a resource than a demand, despite pressure that results from being responsible for other people’s lives.

*‘Working with patients is, in general, not that stressful. I don’t need to prove in front of the patients. I just ask them about their health status and they notice that I want to help them and are glad to cooperate with me.’ (S1, f, 24 yrs, 6*^*th*^*year)*

By setting clear goals, drawing on intrinsic motivation, realizing that the internships are only temporary and dreaming of a less stressful future, for instance, in a specialty that allows a better work-life balance or because they have more experience, students work their way through the stress they experience during the internships. Differences between specialties are mentioned:

*‘I think the job is very stressful and sometimes I’m asking myself ‘How am I going to do that later when I want to have children beside such a job?!’ I don’t know, I hope I can do it part-time, but I don’t know. Yeah, for that reason I think I am going to be a general practitioner, because I like internal medicine and general practice, but internal medicine is a lot more work than general practice’. (S8, f, 23 yrs, 5*^*th*^*year)*

General practice has the reputation of being more relaxed and being able to better accommodate combinations of work and care. However, this seems to be more acceptable for and accepted by women. The following interviewee’s answer reflects gender norms about part-time work, which is widely accepted and supported by the state in the Netherlands:

*‘I would like to work part-time on the one hand, but I am also realistic. I think that’s not really an option for many specialties because there are already so many women in medicine and a substantial number of them want to work part-time and that creates problems for health care. And, well, it also is more accepted if a man works the whole week. I think that it is also expected from you to work fulltime’. (S9, m, 24 yrs, 6*^*th*^*year)*

Some interviewees do not mention having observed stress among supervisors, whereas others do:

*‘I notice that almost all physicians in the hospital are too stressed, and as an intern you feel almost obliged to work as hard as you can and deliver 200% instead of the normal 100%, the norm is really high’. (S12, f, 23, 5*^*th*^*year)*

Medical staff hardly pay attention to the students’ experience of stress, according to the interviewees. The interviewees accept being left alone with their distress because otherwise it might influence their future careers:

*‘So it’s not like somebody says ‘Oh, I see you’re stressed. Why?’ I never heard somebody said that, maybe it’s not important enough. Like if you can’t handle it, you shouldn’t do it’. (S4, f, 22 yrs, 4*^*th*^*year)*

*‘You won’t go there* [to your supervisors] *with your problems, because then their perception of you gets influenced’. (S9, m, 24 yrs, 6*^*th*^*year)*

The norm to remain silent at work about problems may explain why many students observe no stress among their supervisors. Interviewees express that they do not feel psychologically prepared for certain internships and would appreciate the opportunity to talk about stressful situations in a professional environment.

*‘There should be really a course, maybe with a psychologist who tells you how to deal with stress and who gives people solutions how to deal with it and not just talking about it once or twice’. (S5, f, 23 yrs, 5*^*th*^*year)*

Students can disclose their emotions and talk about their stress with friends and family members, which makes it an entirely private matter, but professional confidentiality inhibits them from speaking about patient-related issues.

*‘Another strategy, which works better, is discussing the things I have experienced at work with friends or with my boyfriend. Usually this is possible, but not always. Besides, I cannot, of course, discuss confidential issues with others, so I can only tell certain aspects of a problem’. (S16, f, 22 yrs, 5*^*th*^*year)*

In the hospital, however, the implicit message to remain silent about experiencing stress during medical training teaches students to cope with stressful situations by engaging in problem-avoiding behavior. In particular, when students feel uncomfortable in relation to their supervisor, they keep silent, do not speak up and make sure they do not stand out because of the fear that the supervisor’s evaluation will negatively affect career options. But this will pass, as expressed in the following remark:

*‘When you are a physician, you have more control over your own work, and how you spend your life. Besides, you’ll have so much experience that, hopefully, at a certain point, you will be able to feel self-confident about your decisions’. (S11, f, 22 yrs, 5*^*th*^*year)*

Over time, so they hope, they will be more self-assured and less stressed, have more experience and work in the specialty of their choice, and a better and easier life awaits them.

## Discussion

Interviewees experience stress and anxiety mainly from having to prove themselves in front of staff and peers (*Show What You know…*), while having to find their own solutions for dealing with stress (*… And Deal With Stress Yourself*). Medical interns face many stressors during their internships, and sources of stress are, for example, high workloads and competition between interns. Despite the many stressors, they feel they cannot be open about their experiences of stress because that might have repercussions for their grades and their future opportunities. Self-confidence and stoicism in the face of high stressors, and fierce competition are experienced as important for interns and physicians‘ self-presentation. And when they find it hard to live up to these expectations, they lower their ambitions, consider a specialty with a more ‘controllable’ lifestyle, or just hope that things will be different after graduation. The relationship between stress and performance measures has been reported in earlier studies [8]. It has been shown that Swedish medical students worry about developing work-related health problems such as burnout, and feel that the system leaves them exhausted [63]. In encounters with staff and peers, a powerful message is to keep silent about your well-being, even though physicians are a high-risk population for stress-related disorders [3,42,64]. Interns feel pressure to present themselves as ‘professional and self-confident’, even when they feel insecure, and this adds to their fear of not being suitable for the profession. Patient encounters seem rather a source of pride and satisfaction, as reported earlier [4,10,65]. The role of the medical faculty in fostering caring competence has also been called into question [66], although an earlier study has shown that 6^th^-year Dutch students are more patient-centered than their 1^st^-year counterparts [67]. Thus, a decline in empathy towards patients is not a given.

Showing what you know while remaining silent about stress may place female students at a threefold disadvantage compared to male students. Firstly, in line with other studies [12,52], also among physicians (see literature review [41]), our interviewees perceive that female medical students worry more and feel more insecure about their capabilities and their performance than male students do. This was the perception of male and female interviewees. Secondly, several female interns we interviewed report feeling uncomfortable with required norms to present a confident self. Feelings of being an impostor occur when high achieving individuals believe they are less intelligent and competent than others believe them to be. In previous studies, such impostor feelings were reported by a higher percentage of female than male students, including medical students [22]. The impostor syndrome has also been the highest predictor of students’ distress, although no gender differences in distress were reported [22]. Female students may be more willing to admit to feeling vulnerable, anxious, stressed, or lacking confidence, even when overall levels are similar [53]. And thirdly, female interviewees report that women like to discuss their insecurities more often. Our findings support the importance of self-efficacy in personal development [51], and may be ascribed to stereotypical gender socialization [12,53]. A recent experimental study has also shown that both male and female students’ average speculation of their future performance in an internship did not differ when the comparison peer (a paper case) of the opposite sex is presented as performing less well [68]. But when the peer of the opposite sex is introduced as performing better or similarly, on average male students estimated their own performance as significantly higher than female students. Stereotypes may play a role in these comparisons. New to our study is that female students tend and are perceived to engage more often in extensive study behavior to compensate for their ‘lower capabilities’. Consequentially, this is a risk factor for higher stress; working more hours is a risk factor for increased work-related fatigue in particular for highly educated women and for burnout in female physicians [13,16]. In turn, stress may raise doubts about their capabilities. Medical education is insufficiently receptive to students’ needs and indeed it fosters silence, while shared reflection reduces stress and improves learning [1,8]. Other studies suggest that this may be important for female physicians in particular e.g. [13]. As early as 1984 in the US, support groups and networks for female faculty and students were formed to provide role models and mentoring [39]. Our study suggests that such measures may still be useful.

Students’ perception of general practice as relatively open for combining work and care has been reported earlier [69,70], but the finding that interns consider general practice to be less stressful is new. Swedish medical students have reported that they are not prepared to sacrifice family life and hobbies although these wishes may not be fulfilled upon their entrance into the profession [63,70,71]. Nevertheless, multiple roles may mitigate strain; having children has repeatedly been shown not to statistically explain stress or even to be a protective determinant for stress in highly educated women including physicians [13,16]. In the Netherlands, many women work part-time, and Dutch female students especially may perceive working part-time as a legitimate way out of stress. However, the high burnout levels among general practitioners, where working part-time is highly common, show that they may be in for an unpleasant surprise about job demands [2,3].

Our findings suggest that, despite the influx of female physicians and students, a ‘patriarchal culture’, which favors aspects such as rationalism, certainty and invulnerability is still experienced by medical students [14]. Students perceive medical culture as hierarchical and competitive in which disclosing feelings of vulnerability and stress has negative consequences for their grades and their opportunities for future specialties. As a result, they hide their feelings of stress as much as possible from supervisors. This resembles Bleakley’s idea of ‘assertive confidence’ as a ‘defence against recognition of uncertainty’ [14]. However, the interns perceive some differences between male and female students in their responses to this possible ‘masculine protest’. For female students, shying away from certain specialties and utilizing part-time work, which is often framed as a way to combine family with work, seems more acceptable. This fits into the ideology of strong Dutch motherhood, where personally taking care of small children is considered the parents’ (read: mothers’) job. However, our findings suggest a second rationale behind many female doctors’ preferences for part-time work. Possibly, this response to medicine’s patriarchal culture can be conceived of as a feminine protest against the masculine protest. This ‘feminine protest’ may be another, and in fact literal, way of distancing one’s self from the medical culture. Besides, we are not certain the ‘masculine protest’ is actually a protection from the suffering which doctors perceive from their patients, because working with patients does not seem to be a major source of stress for interns. Hence, the ‘masculine protest’ may rather be a protection from a professional culture in which it is hard to acknowledge vulnerability and uncertainty as a physician. Both the ‘masculine’ and the ‘feminine protest’ are presented as individual responses to a professional culture perceived as a given, as shown in some of the quotes. Therefore, the ‘protesting’ is still rather conformist.

The Effort-Recovery Model explains high stress levels from discrepancies between job demands, job control and work potential [43]. Our results do provide evidence for the model, for instance, because the internships seem to require a lot of effort from the students, with long working weeks, studying hard and showing that they are motivated and knowledgeable, and the students relate these aspects to feelings of stress. Our findings suggest that job demands are high for all interns, while job control is rather low. However, our study also suggests that gendered aspects as well as feelings of stress play roles in the assessment of work potential itself, by students and by their peers. Hence, stress is not necessarily an *outcome of* a discrepancy, it can also be *the source of* such perceived discrepancy. The Effort-Recovery Model seems to ignore the gendered dimensions of: (a) *recovery*, which may apply better to male students’ (who are perceived to seek recovery by taking leisure time, doing sports) stress responses; and (b) *cumulation*, which may apply better to female students’ (who are perceived to intensify their efforts by studying harder which may further impair their general well-being) responses. When female interns have done everything within their power to increase their work potential, yet still experience stress as a result of discrepancy between their capabilities and work demands, then aiming to work fewer hours in a less demanding specialty may seem like a sensible strategy. However, such a strategy is also considered as evidence that they are not completely suitable for the profession, and thus, have lower work potential. This may be one explanation for the persistent gender segregation in the profession [19].

### Methodological considerations and future research

There are a number of strengths and limitations to the current study. Firstly, the study is retrospective and later experiences may modify earlier experiences of stress. We also have no information about the particular internships the students were following when they were interviewed, and their current position may have influenced their reports. However, students from all internship years were interviewed, and thus we have sufficient variation. Secondly, qualitative work ideally requires fluency in the language, which was not the case in this study. The first interviewer had a German background and conducted the interviews in English. This may have attracted students who were proficient in English and nuances may have been lost, although both Dutch and German students have profound training in English during their education from an early age. The fact that both the interviewer and interviewees were not native speakers of English may have enhanced relationship building during the interviews, but it may also have been an obstacle for communication. The first series of interviews was also analyzed in English. The second series of interviews was conducted and analyzed in Dutch, and quotes were translated to English afterwards. And although we saw no evidence for lacking nuances or possibly contradictory findings, language issues still may have influenced the results [59]. Thirdly, both interviewers were health sciences students. Interview training was part of their graduate programme, but neither of them was an experienced interviewer. This is possibly correlated with the finding that the interviews provide a good insight into ‘daily’ stressors and problems, but not into highly problematic and sensitive topics such as harassment. And finally the number of interviewees was small, and the qualitative approach does not allow for universal conclusions. Among the 17 interviewees only six were men, which is a relatively low number. Nevertheless, both female and male students perceive similar gendered patterns in regards to dealing with stress by themselves and their colleagues. Variation in medical students’ perception of own stress was revealed. Therefore, data and theoretical saturation have been achieved [59,62], in particular, regarding a gendered perspective. Our initial purposive recruitment approach ended up in being one of convenience. However, the interviewees report that they prefer discussing stress with friends in a private sphere. Hence, the fact that interviewees were found through personal contacts may actually have benefited disclosure to the interviewer.

Qualitative and quantitative research have their own merits. A quantitative study on stress among interns based on the Effort-Recovery Model may provide insight into students’ experience of stress, factors that are associated with stress and gender differences therein. The qualitative approach allows us to incorporate a more fluid gendered perspective and to reflect on the perceptions and processes of (doing) gender during the internships. Future research, both quantitative and qualitative, may include a greater variety of medical students and locations (in order to study differences in the experience of stress across wards), or include a particular focus on students with an ethnic minority background. In a US study, ethnic mistreatment of students was high, despite institutional efforts to reduce it, and it was found to not likely be reported by students [33]. Investigating residents and supervisors’ attitudes towards stress, as well as gender and ethnic differences therein may provide further tools for improvement of medical education in the clinical years. Most importantly, studying the consequences for male and female students and physicians of detaching oneself not only from patients, but in particular from *their own feelings* of insecurity in order to adhere to current professional norms is important. This could help to understand the gendered dynamics behind the paradox that physicians should compromise their own health to the benefit of their patients just because that is considered more professional. With Bleakley, we advocate that medical education welcomes a democratizing process in which collaborative teamwork with colleagues and patient-centered collaborations are the means to improve patient care [14].

## Conclusions

Interviewees experience stress because they have to show what they know, while having to deal with stress themselves. They feel they must present themselves as ‘professional and self-confident’ and remain silent about experiencing stress. Female students may face more disadvantages by this culture. Raising awareness among supervisors about their impact on students’ stress is important. Medical students may benefit from a clearer picture of different specialties and the expectations of their members both during and after medical training. Communication and confidential discussions at work within departments, mentoring and support from superiors are protective determinants of doctors’ well-being [8,42,63,64,72]. Our findings suggest that such measures may particularly benefit those who feel less able to manifest the masculine protest, more often female students and physicians. The culture in the hospital, as well as professionals’ health, will benefit from an open conversation about stress and the creation of an emotionally secure context for learning [21,24,63]. Implementing such a culture might, for instance, be accelerated by producing student evaluations of the openness towards stress and of the (dis)respect that they have encountered during their rotations [33]. All specialties must accommodate to their future members’ needs, male and female and across groups, to safeguard physicians’ well-being and consequentially, patient care.

## Competing interests

The authors declare that they have no competing interests.

## Authors’ contributions

PV, VR and RdV have designed the study. VR and RdV have recruited interviewees and conducted the interviews. All authors were involved in the coding process. PV and IH drafted the article, which was revised for important intellectual content by all authors. All authors have approved the final manuscript.

## Pre-publication history

The pre-publication history for this paper can be accessed here:

http://www.biomedcentral.com/1472-6920/14/96/prepub
